# The sulfiredoxin-peroxiredoxin redox system regulates the stemness and survival of colon cancer stem cells

**DOI:** 10.1016/j.redox.2021.102190

**Published:** 2021-11-15

**Authors:** In-Sung Song, Yu Jeong Jeong, Yena Jung, Young-Ho Park, Sungbo Shim, Sung Joo Kim, Dae-Woon Eom, Seung-Mo Hong, Peter C.W. Lee, Sun-Uk Kim, Sung-Wuk Jang

**Affiliations:** a Department of Biomedical Sciences, College of Medicine, Ulsan University, Asan Medical Center, Seoul, 138-736, Republic of Korea; b Futuristic Animal Resource &Research Center, Korea Research Institute of Bioscience and Biotechnology, Chungchenongbuk-do, 28116, Republic of Korea; c Department of Functional Genomics, KRIBB, School of Bioscience, Korea University of Science and Technology, Republic of Korea; d Department of Biochemistry, College of Natural Sciences, Chungbuk National University, Cheongju, Republic of Korea; e Department of Pathology, Asan Medical Center, University of Ulsan College of Medicine, Seoul, 138-736, Republic of Korea; f Department of Pathology, Gangneung Asan Hospital, Gangneung, Republic of Korea; g Asan Medical Institute of Convergence Science and Technology, Asan Medical Center, University of Ulsan College of Medicine, Seoul, 138-736, Republic of Korea; h Department of Biochemistry and Molecular Biology, University of Ulsan College of Medicine, Seoul, 138-736, Republic of Korea

**Keywords:** Colon, Cancer stem cell, Sulfiredoxin, Peroxiredoxin, Stemness, ROS, OXPHOS, CSC, cancer stem cell, DMEM, Dulbecco’s modified Eagle’s medium, FACS, fluorescence-activated cell sorting, KI, knock-in, MACS, magnetic-activated cell sorting, OCR, oxygen consumption rate, OXPHOS, oxidative phosphorylation, PBS, phosphate-buffered saline, Prx, peroxiredoxin, ROS, reactive oxygen species, Srx, sulfiredoxin, WT, wild-type

## Abstract

Cancer stem cells (CSCs) initiate tumor formation and are known to be resistant to chemotherapy. A metabolic alteration in CSCs plays a critical role in stemness and survival. However, the association between mitochondrial energy metabolism and the redox system remains undefined in colon CSCs. In this study, we assessed the role of the Sulfiredoxin-Peroxiredoxin (Srx-Prx) redox system and mitochondrial oxidative phosphorylation (OXPHOS) in maintaining the stemness and survival of colon CSCs. Notably, Srx contributed to the stability of PrxI, PrxII, and PrxIII proteins in colon CSCs. Increased Srx expression promoted the stemness and survival of CSCs and was important for the maintenance of the mitochondrial OXPHOS system. Furthermore, *Nrf2* and *FoxM1* led to OXPHOS activation and upregulated expression of Srx-Prx redox system-related genes. Therefore, the *Nrf2/FoxM1*-induced Srx-Prx redox system is a potential therapeutic target for eliminating CSCs in colon cancer.

## Highlights

• CSCs initiate tumor formation and are known to be resistant to chemotherapy.
• We assessed the role of the Srx-Prx redox system and OXPHOS in colon CSCs.
• Srx contributed to the stability of PrxI, PrxII, and PrxIII proteins in colon CSCs.
• Nrf2 and FoxM1 upregulated Srx-Prx redox system-related gene expression.
• Nrf2/FoxM1-induced Srx-Prx redox system is a target to eliminate CSCs in colon cancer.

## Graphical abstract

**Figure:**
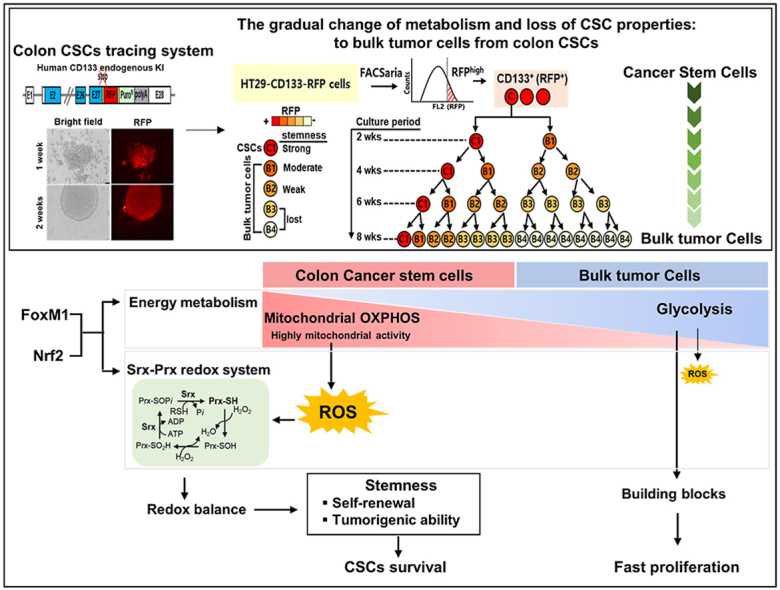

## 1 Introduction

Cancer stem cells (CSCs) are well-defined as a small cell population within a tumor, with the capacity to initiate tumor formation and undergo extensive proliferation, and are reportedly resistant to chemotherapy [1,2]. Additionally, CSCs express stem cell-specific markers and are maintained via asymmetric self-renewal [3]. These properties of CSCs result in tumor relapse, therapy resistance, and metastasis, leading to poor prognosis in patients. Hence, it is crucial to assess the characteristics of CSCs, which could facilitate the development of strategies for targeted therapy to eliminate CSCs. Previous studies have reported similarity between CSCs and stem cells; for example, CSCs are reportedly quiescent cells with low mitochondrial activity that utilize glycolysis to generate adenosine triphosphate (ATP) [4,5]. However, we have recently demonstrated that colon CSCs utilize the mitochondrial oxidative phosphorylation (OXPHOS) system to generate ATP and increase reactive oxygen species (ROS) levels when compared to non-CSCs. Moreover, colon CSCs potentially utilize primary OXPHOS rather than glycolysis to produce ATP [6]. Notably, colon CSCs exhibit increased mitochondrial function compared to colon bulk tumor cells [7,8]. Furthermore, a small population of pancreatic ductal adenocarcinoma cells has been previously identified as pancreatic CSCs due to the conversion of the carbon source from glucose to galactose and the utilization of the OXPHOS system [9].

Cancerous cells undergo metabolic reprogramming to meet their increased energy requirements caused by continuous growth, rapid proliferation, and other characteristics typical of neoplastic cells [10,11]. Such an alteration in metabolism involves several steps, including aerobic glycolysis, glutamine catabolism, macromolecular synthesis, and redox homeostasis to support the rapid growth of cancerous cells. Such metabolic modifications evolve as the CSCs within cancer progress through various stages, including the tumor initiation stage with CSCs, localized tumor progression, and distant metastasis. However, tumors observed in humans are metabolically heterogeneous [12]. Although a tumor may develop in the same tissue, tumor cells may differ in their energy metabolism mechanisms (such as mitochondrial OXPHOS or glycolysis) to produce ATP, depending on their metabolic adaptation [11,13]. Additionally, tumor cells’ metabolic reprogramming may be accompanied by change in the redox system [14,15].

Numerous cancers, including colon cancer, rely on the redox system for survival [15]. ROS not only regulate the stemness of hematopoietic stem cells but also strongly influence pluripotency [16,17]. Sulfiredoxin (Srx) is an antioxidant enzyme that primarily reduces the hyperoxidized 2-Cys Peroxiredoxin (Prx) reversal to active peroxidase in an ATP-dependent manner and then reduces the excessive ROS levels to protect the cells from oxidative stress [15]. In the catalytic cycle of 2-cys Prx, 2-cys Prx can oxidize the thiol group of the cysteine (Cys-SH) to sulfenic acid (Cys-SOH), which may then react with the resolving Cys-SH to form a disulfide bond (Cys-S-S-Cys). During more severe oxidative stress, the sulfenic intermediate can be further oxidized to sulfinic acid (Cys-SO_2_H) or sulfonic acid derivatives (Cys-SO_3_H) [18,19]. The hyperoxidation reaction results in the inactivation of peroxidase function [19].

In the present study, we investigate the association between mitochondrial energy metabolism and the redox system in colon CSCs.

## 2 Materials and methods

### 2.1 Cell culture, antibodies, and chemicals

Human colon cancer cell lines HT29, HCT116, SW480, and SNU-C5 were obtained from the Korean Cell line Bank (Seoul, Korea). HT29, HCT116, SW480, and SNUC5 cells were cultured in Dulbecco's modified Eagle's medium (DMEM) supplemented with 10% fetal bovine serum (FBS). All cell cultures were incubated at 37 °C in a 5% CO_2_ humidified incubator. The primary antibodies used included Poly-(adenosine diphosphate-ribose) polymerase (Cell Signaling Technology, Danvers, MA, USA), Phospho-histone H2A.x (S139; Cell Signaling Technology), PrxI, PrxII, PrxIII, and Prx-SO_2_/_3_ (all from Abclone, Seoul, Korea), Srx, cIAP-1, and Tubulin (all from Santa Cruz Biotechnology, Dallas, TX, USA), and CD133 (including CD133 antibodies conjugated to magnetic beads and APC; Miltenyi Biotec, Auburn, CA, USA). The Srx inhibitor frugoside was isolated from *Cardiospermum halicacadum*, as previously described [20].

### 2.2 Tissue collection from colon cancer patients

Human tissues were obtained in accordance with the ethical standards of the Institutional Review Board for Human Research at Inje University Busan Paik Hospital (approval number: 15–0081). Samples were obtained from six patients (age range, 30–72 years) who underwent surgery to treat colon adenocarcinoma. The tumor features and case description of samples isolated from six patients are presented in Supplementary Table 2. The colon cancer tissues were washed intensively in phosphate-buffered saline (PBS) solution twice, after which enzymatic digestion was performed using collagenase (1.5 mg/mL) and hyaluronidase (20 mg/mL; Sigma, St Louis, MO, USA) in PBS for 1 h. The isolated cells were then sorted using Fluorescence Activated Cell Sorting (FACS) into CSCs and non-CSCs.

### 2.3 FACS, magnetic-activated cell sorting, and flow cytometry

Cells from the human colon cancer cell lines, HT29, HCT116, and SNU-C5, were resuspended, with PBS, and stained with the CD133/1-APC antibody. The stained cells were then sorted using a BD FACSAria flow cytometer (Becton Dickinson, Franklin Lakes, NJ, USA). Magnetic-Activated Cell Sorting (MACS) was performed on the tumor cell populations using cell isolation kits containing microbeads conjugated with a CD133/1-specific antibody. The magnetic separation step was repeated twice using positive (LS column) and negative selection columns (LD column). After magnetic sorting, cell viability was assessed using trypan blue exclusion. The quality of separation was determined using flow cytometry with an antibody against CD133/2 (Miltenyi Biotech) for both the CD133^+^ and CD133^-^ cell populations.

### 2.4 Generation of the *Srx* knockdown cell line and TagRFP reporter for CRISPR/Cas9 genome editing at the *CD133* locus

To generate a stable *Srx* knockdown, the single guide RNA (sgRNA) sequence targeting the *Srx* gene was subcloned into Tet-On 3G all-in sgRNA-Cas9 plasmids (ToolGen, Korea), which were used to transfect HT29 cells. For the knockout validation, genomic DNA from selected clones was sequenced after amplifying the nearby DNA sequence complementary to the *Srx* gene sgRNA. A TagRFP reporter was designed for CRISPR/Cas9 genome editing at the *CD133* locus, as shown in Supplementary Fig. 1. The constructed cell lines were named HT29-SrxKO and HT29-WT, and represented as SrxKO and WT in all figures. The plasmid pCAG-SpCas9-GFP-U6-gRNA (plasmid #79144; Addgene) was used to express the sgRNA. An analysis of the C-terminal coding region of *CD133* revealed several potential Cas9 guide sites in exon 27, which were identified as having high target affinities, high efficiencies, and low off-target scores using online CRISPR/Cas9 sgRNA design tools (http://www.rgenome.net/cas-offinder/and http://dash.harvard.edu/handle/1/13581017). The optimal scoring guide target site [GGG]GCTATCAATGTTGT-GATACT (PAM site indicated in brackets) was selected for CRISPR gene-editing via Cas9. A C-terminal donor around the Cas9 cleavage site was designed to replace the TGA stop codon of CD133 with a 2A peptide insert, comprising a TagRFP-E2A-TagRFP-T2A-Puro^R^-bGH poly(A) cassette surrounded by 1.5-kb (left) and 2-kb (right) flanking arms that were homologous to the locus. The insert was designed to remain in-frame with *CD133*, while the 2A peptide ensured the expression of the TagRFP reporter and the native *CD133* gene as separate proteins.

### 2.5 Measurement of oxygen consumption rate and extracellular acidification rate

To measure the OCR and the extracellular acidification rate (ECAR), we used an XF24 Extracellular Flux Analyzer (Seahorse Bioscience, Billerica, MA, USA). Briefly, colon cancer cell lines were sorted into CD133^+^ and CD133^-^ subpopulations and plated at a density of 2 × 10^4^ cells/well in a Corning Cell-Tak (Corning, Bedford, MA, USA)-treated XF24 cell culture plate (Seahorse Bioscience). The plate was centrifuged at 200×*g* for 3 min to remove the medium. Subsequently, 500 μL of XF assay medium (modified DMEM, Seahorse Bioscience) was added, and the plate was incubated at 37 °C without CO_2_ for 1 h. The OCR was measured using the Mito Stress application, and the ECAR was measured using the Glycolysis application of the XF24 analyzer software.

### 2.6 Measurement of ROS production

ROS production was assessed using the ROS indicator 5,6-chloromethyl-2′,7′-dichlorodihydro-fluorescein (CM-H_2_DCFDA; Thermo Fisher Scientific, Waltham, MA, USA). The cells were seeded on 35-mm dishes and incubated for 24 h. The cells were then starved of serum for 6 h and cotreated with TNFα plus CHX in phenol red free media. The cells were rinsed once with 2 ml KREB's Ringer bicarbonate buffer and incubated for 5 min with CM-H_2_DCFDA. The dishes were mounted, and immediately analyzed under fluorescence microscopy (Zeiss Axiovert 200 M).

### 2.7 Immunohistochemistry analysis

Patient tissues were fixed with 10% formalin, embedded in paraffin, and processed into 5-μm-thick sections. The paraffin sections were immunostained with anti-Srx, anti-CD133, anti-Prx I, anti-Prx II, and anti-Prx III antibodies according to the manufacturer's protocol (DAKO, Carpinteria, CA, USA). The stained sections were examined using an Olympus BX51 microscope, and images were acquired using an Olympus DP70 camera (Olympus, Tokyo, Japan).

### 2.8 Transfection of siRNA against *Srx* gene and real-time polymerase chain reaction

For *Srx* knockdown, an siRNA sequence (5ʹ-GGAGGUGACUACUUCUACU-3ʹ) with >80% *Srx* knockdown efficiency was constructed based on an *Srx* sequence. Total cellular RNA was extracted from human colon tumor tissues, non-tumor tissues, colon cancer cell lines, and sorted CD133^+^/CD133^–^ cells using an RNA extraction kit (Qiagen, Tokyo, Japan). RNA (1 μg) was reverse-transcribed using a First-Strand cDNA Synthesis kit (Fermentas, Grand Island, NY, USA). qRT-PCR was performed using SYBR Green in an ABI 7900HT Fast Real-Time PCR System (Applied Biosystems, Foster City, CA, USA). All reactions were performed in triplicate, and beta-2-microglobulin (*B2M*) or GAPDH was used as the housekeeping gene. Using the comparative threshold cycle method or standard method, relative *Srx* gene expression was quantified by determining the ratio of *Srx* expression in CD133^+^ to CD133^–^ cells after normalization against *B2M* expression in each sample. The primer sequences used in the present study are listed in Supplementary Table 1.

### 2.9 Colony-forming assay and sphere-formation assay

To evaluate the tumorigenicity of the cells, colony-forming and sphere-formation assays were performed in soft agar and ultra-low-attachment six-well plates, respectively. For the colony-forming assays, the cells were suspended in 1 mL of cell growth medium containing 0.3% agar and plated over a layer of 0.6% agar in the growth medium. The plated cells were grown for 15 days, after which the colonies were stained with 0.01% crystal violet (Sigma) for 10 min and counted. For the sphere-formation assay, the cells were seeded in ultra-low-attachment six-well plates at a density of 10^4^ cells/well. Spheroid cultures were grown in serum-free DMEM/F12 medium supplemented with 4 mg/mL bovine serum albumin, 10 ng/mL basic fibroblast growth factor, 20 ng/mL epidermal growth factor, 100 μg/mL apotransferrin, and 25 μg/mL insulin for a week.

### 2.10 Cell death and determination of morphological changes using TMRE/Hoechst 33342

*Srx*-silenced human colon cancer cell lines were treated with 5-FU for 24 h at the indicated doses. For cell death experiments, *Srx*-silenced HT29 cells were cultured with TNFα (20 ng/mL) and cycloheximide (CHX, 10 μg/mL) for the indicated times. Similarly, stable *Srx*-depleted cells were treated with 5-FU or TNFα/CHX. Cell death was measured using flow cytometry after staining the cells with an annexin V-fluorescein isothiocyanate/propidium iodide (Annexin V-FITC/PI) staining kit (Roche, Nutley, NJ, USA). Hoechst 33342 and TMRE dye were used to stain the nuclear chromatin and mitochondrial membranes, respectively, and determine the morphological changes caused by Srx depletion or TNFα/CHX treatment.

### 2.11 Comet assay

Alkaline comet assays were performed using a comet assay kit (Cell Biolabs, San Diego, CA, USA), according to the manufacturer's protocol. The cells were mixed with low-melting agarose gel and spread on the comet slide. Subsequently, the cells were lysed on ice with the lysis solution and then equilibrated with an alkaline electrophoresis buffer. Images of the slides were obtained using a fluorescence microscope. The tail streaks of 50 randomly selected cells were measured and averaged.

### 2.12 Protein isolation and western blotting

The cells were lysed in lysis buffer A (20 mM N-2-hydroxyethyl piperazine-Nʹ-2-ethanesulfonic acid [pH 7.5], 150 mM NaCl, 1 mM EDTA, 2 mM ethylene glycol tetraacetic acid, 1% Triton X-100, 10% glycerol, and protease cocktail I/II; Sigma). Cell debris was removed by centrifugation at 10,000 × *g* for 10 min. The proteins were separated using sodium dodecyl sulfate–polyacrylamide gel electrophoresis and transferred to nitrocellulose membranes. The membranes were then blocked with 5% skimmed milk in 0.01 M TBS (pH 7.5) containing 0.5% Tween 20 and blotted with the appropriate primary antibodies. The antigen–antibody complexes were detected using chemiluminescence (Abclone, Korea).

### 2.13 Measurement of mitochondrial activity and ATP production

For the detection and measurement of mitochondrial membrane potential, mitochondrial ROS, and calcium concentration, we used the specific fluorescent probes TMRE, Mito-Sox, and Rhod-2AM (Thermo), respectively. Colon cancer cells were cultured and incubated with 5 μM TMRE and Rhod-2AM for 30 min and 1 μM Mito-Sox for 20 min at 37 °C. The fluorescence levels were then measured using a FACSCanto™ II flow cytometer (BD Biosciences). To measure and analyze ATP levels, we used an ATP determination kit (Thermo Fisher Scientific, Waltham, MA, USA) according to the manufacturer's protocol. Briefly, CD133^+^ and CD133^-^ cells were sorted from colon cancer cell lines. The cells were plated at a density of 2 × 10^5^ cells/well in a six-well plate and added to 2 ml of fresh medium (10 mM glucose in FBS-free DMEM/F12) for the total ATP level measurements. The plate was incubated at 37 °C in a humidified incubator for 90 min. The cells were harvested, lysed, and subjected to an ATP determination assay. The ATP levels were measured in each well after adding an ATP detection agent, and the results were quantified using a luminometer (Molecular Devices, Sunnyvale, CA, USA). To measure the mitochondrial ATP and total ATP production, we used an XF24 analyzer (Seahorse Bioscience). Briefly, the cells were plated at a density of 2 × 10^4^ cells/well in a XF24 cell culture plate (Seahorse Bioscience). One well is incubated in 500 μl XF Assay medium-modified DMEM (10 mM glucose in FBS-free DMEM), and another well is incubated in mitochondrial inhibitor-containing medium (10 mM galactose, 1 μM rotenone, and 1 μM antimycin A) to block mitochondrial electron transport chain complex I and III activity. The plate was incubated at 37 °C in a humidified incubator for 90 min and was measured the glycolysis application of the XF24 software. The total Cellular ATP production rate is the sum of the glycolytic and mitochondrial ATP production rates: mitochondrial ATP production (pmol ATP/min) = total ATP production (pmol ATP/min) - glycoATP production (pmol ATP/min).

### 2.14 Evaluation of tumorigenicity and toxicity

Tumorigenicity was determined by subcutaneously injecting HT29 WT and HT29-*Srx*KO CD133^+^ cells (5 × 10^5^) isolated by MACS into the flanks of six-week-old female nude mice. All institutional and national guidelines for the care and use of laboratory animals were followed (No.2018-12-217). All animal procedures were approved by the Institutional Animal Care and Use Committee of the Asan Institute for Life Sciences (Republic of Korea). After 10 d, well-established tumors with approximate volumes of 100 mm^3^ were detected. Four groups (HT29 WT, HT29 WT– 5FU, HT29-Srx KO, and HT29-Srx KO − 5FU) were formed by pooling three mice from each of the previous groups. The experimental mice were intraperitoneally injected with 5-FU (50 mg/kg/day, three days/week for two weeks), while control mice were injected with PBS. The tumor size was measured every two days using a digital caliper. The tumor volumes (V) were calculated from the measured length (l) and width (w) using the following formula:

V = lw^2^/2.

The mice were sacrificed 28 d after the injection, and tumor samples were analyzed using the DeadEnd Colorimetric TUNEL System (Promega, Madison, WI, USA). For tumor transplantation into the cecum, the mice were first anesthetized by inhalation of isoflurane, and a small nick was then made in the skin. The cecum was exteriorized, and the cecal wall was slightly damaged. The tumor piece originating from HT29-WT or HT29-*Srx*KO was then positioned and tied down. The mice were euthanized at 10 weeks post-transplantation to assess the tumor burden or number.

### 2.15 Statistical analysis

Statistical analysis was performed using the Student's *t*-test and SigmaPlot 12.0 (Systat Software Inc, San Jose, CA, USA). Statistical significance was defined as follows: *, *P* < 0.05; **, *P* < 0.01; and ***, *P* < 0.001. All the data in the study are expressed as the mean ± standard deviation obtained from the results of three independent experiments.

## 3 Results

### 3.1 Preferential utilization of OXPHOS in colon CSCs accompanied by an alteration in the redox balance system

**Fig. 1:**
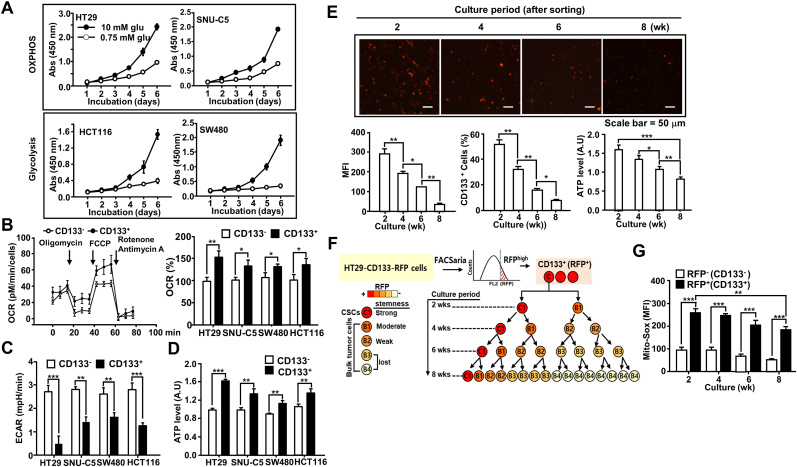
**Colon cancer stem cells (CSCs) utilizing the oxidative phosphorylation (OXPHOS) system differentiated into glycolysis-dependent bulk tumor cells. (A)** Colon cancer cell lines were categorized as the OXPHOS group and glycolysis group, based on their proliferation rate in 0.75 mM low-glucose and 10 mM high-glucose media. Abs, Absorbance. **(B–C)** The difference in oxygen consumption rate (OCR) (B) and lactic acid production (C) was measured in CSCs and non-CSCs isolated from the CD133^+^ and CD133^–^ cells in each cell line. **(D)** ATP levels were determined by quantifying the luciferase-catalyzed, ATP-dependent oxidation of luciferin in CSCs and non-CSCs. **(E**–**G)** HT29-CD133-RFP cells were sorted to isolate the RFP^high^ CSCs. The cells were then cultured for eight weeks. (E) CD133-RFP expression was assessed in the sorted RFP^+^ cells every two weeks. The upper panel demonstrates the decline in RFP expression in cultured cells over time. The changes in CSC population (RFP expression, lower left; percent of CD133^+^ cells, middle panels) and ATP level (lower right panel) were also assessed. MFI, mean fluorescence intensity. Scale bar: 50 μm (F) Schematic illustration of the time-associated change in bulk tumor cells generated from CSCs, resulting in the loss of the stemness characteristic in these cells. (G) The cultured HT29-CD133-RFP cells at each time point were sorted into RFP^+^ cells and RFP^–^ cells. The cells were then stained with a Mito-SOX dye, and the mitochondrial reactive oxygen species (ROS) level was measured using FACS analysis. Statistical significance was defined as follows: *, *P* < 0.05; **, *P* < 0.01; and ***, *P* < 0.001. All the data in the study are expressed as the mean ± standard deviation obtained from the results of three independent experiments.

To define the characteristics of CSCs present within the tumor bulk that were altered by metabolic reprogramming, we used four colon cancer cell lines. We divided them into a mitochondrial OXPHOS system-dependent group and a glycolysis-dependent group. The sorting was performed according to the differential growth rate of the cells in culture media containing varying glucose concentrations (0.75 and 10 mM). Many cancer cells undergoing metabolic reprogramming become glycolysis-dependent cells that consume increased glucose levels for the generation of ATP and building blocks [[21], [22], [23]]. Recently, Birsoy et al. reported that cancer cells exhibit diverse responses to glucose limitation and identified defects in glucose utilization and mitochondrial function as major determinants of low-glucose sensitivity [23]. The HT29 and SNU-C5 cell lines exhibited similar growth rates in both the 0.75 and 10 mM glucose conditions (OXPHOS-dependent group), while the growth rates of SW480 and HCT116 cells decreased when cultured in 0.75 mM glucose media (glycolysis-dependent group; Fig. 1A).

We then assessed the energy-producing system of CSCs in each cell line. The CSCs from each cell line were isolated, and the oxygen consumption rate (OCR), lactic acid production, and mitochondrial ATP level in colon CSCs and non-CSCs isolated from each cancer cell line were measured. The OCR and ATP level in CSCs, regardless of the original cell group (glycolysis- and OXPHOS-dependent group), were higher than those in non-CSCs, whereas lactic acid production was higher in non-CSCs (Fig. 1B – D). Collectively, the results suggest that the metabolic alteration of bulk tumor cells derived from CSCs facilitated varying energy-producing systems, including glycolysis-dependent systems or OXPHOS-dependent systems, in colon cancer. Moreover, the alteration in bulk tumor cells may have gradually led to the acquisition of a glycolysis-dependent cell state from OXPHOS-dependent cells.

To trace the gradual change in metabolism in bulk tumor cells resulting from CSCs, we generated an HT29-CD133-RFP cell line by inserting an RFP sequence with the 2A self-cleaving peptide at the stop codon of the gene encoding CD133 in CSCs using the CRISPR/Cas9 knock-in system (Supplementary Fig. S1). The HT29-CD133-RFP cells are isolated into CD133-low-expressing cells (RFP-negative population) and CD133 high-expressing cells (RFP-positive population) according to levels of RFP expression using a BD FACSaria flow cytometer (Becton Dickinson). Cells were then cultured for two months, after which CD133 expression and cellular ATP levels were confirmed. With prolonged culture, RFP-expressing cells were altered to non-RFP-expressing cells (CD133^-^) from high RFP-expressing cells (CD133^+^), as well as original isolated CSCs (RFP^+^) (Fig. 1E). Moreover, the mitochondrial ATP levels in the high RFP-expressing cells decreased with prolonged culture periods (Fig. 1E). Thus, we propose that the bulk tumor is generated by CSCs that undergo asymmetric cell division along with loss of their characteristic stemness (Fig. 1F). Furthermore, the results indicated that colon CSCs preferentially utilized the OXPHOS system to generate ATP, while the differentiated bulk tumor cells gradually switched to the glycolysis-dependent system. Additionally, our results suggested that ATP generation by the mitochondrial OXPHOS-employing CSCs may be accompanied by high ROS production. We confirmed that ROS levels were higher in colon CSCs than in non-CSCs (Fig. 1G). Therefore, we concluded that to maintain the stemness of colon CSCs, coordinated action between the energy metabolism system and redox system is required to eliminate high ROS levels.

### 3.2 Energy production via the mitochondrial OXPHOS system alters the Srx-Prx redox system in colon CSCs

**Fig. 2:**
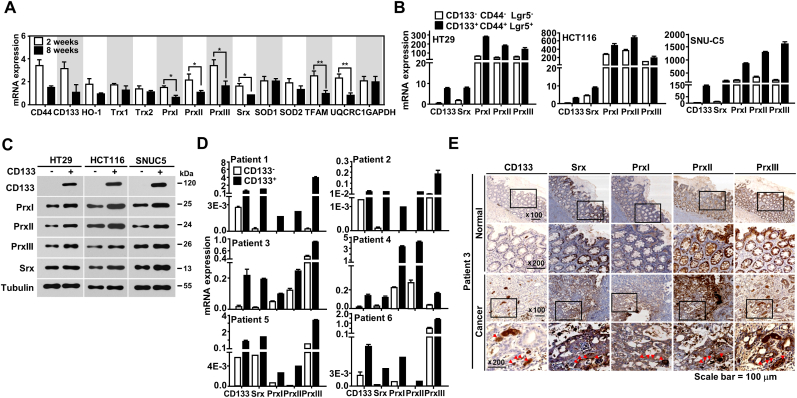
**Upregulated expression of sulfiredoxin (Srx), peroxiredoxin (Prx) I, PrxII, and PrxIII in CSCs. (A)** Real-time polymerase chain reaction (qRT-PCR) was used to analyze the expression of genes related to antioxidant and mitochondrial function in cells cultured for two and eight weeks with RFP^+^ cells sorted from HT29-CD133-RFP cells. **(B–C)** The mRNA (B) and protein levels (C) of Srx, PrxI, PrxII, PrxIII, and CD133 in cells isolated from the colon cancer cell lines HT29, HCT116, and SNUC5 using antibodies specific for CD133, CD44, and Lgr5 were analyzed using qRT-PCR and immunoblotting. **(D)** The mRNA expression of *Srx, PrxI, PrxII, PrxIII,* and *CD133* in CD133^+^ and CD133^–^ cells isolated from colon cancer patient samples were analyzed using qRT-PCR. Gene expression was normalized against GAPDH mRNA expression in each sample. **(E)** The expression of Srx, PrxI, PrxII, PrxIII, and CD133 proteins in colon cancer tissues and adjacent normal tissues was evaluated using immunohistochemistry. Red arrows indicate highly positive cells. Scale bar = 100 μm. Statistical significance was defined as follows: *, *P* < 0.05; **, *P* < 0.01. All the data in the study are expressed as the mean ± standard deviation obtained from the results of three independent experiments. (For interpretation of the references to colour in this figure legend, the reader is referred to the Web version of this article.)

To examine whether the antioxidant system for ROS elimination was altered in colon CSCs, we first measured the levels of various antioxidant genes in the short-term (2 weeks) and long-term (8 weeks) culture groups isolated from HT29-CD133-RFP cells. Srx-Prx redox system-related genes were highly expressed in the short-term compared to in the long-term culture group (Fig. 2A). To determine whether the Srx-Prx redox system could maintain the stemness of colon CSCs and facilitate survival, we subsequently analyzed Srx and Prxs expression in colon CSCs. We observed increased *Srx* and *Prxs* mRNA expression in CSCs sorted using CD133, CD44, and Lgr5 antibodies relative to non-CSCs in several colon cancer cell lines (Fig. 2B). Immunoblotting analysis confirmed that Srx and Prxs protein expression was upregulated in CD133^+^ CSCs isolated from the HT29, HCT116, and SNUC5 colon cancer cell lines (Fig. 2C). We also observed significantly increased *Srx*, *PrxsI*, *PrxII,* and *PrxIII* expression via quantitative real-time polymerase chain reaction (qRT-PCR) in CD133^+^ CSCs freshly isolated from colon cancer patients (Fig. 2D). Immunostaining for Srx, Prxs, and CD133 was performed on tissue sections obtained from patients with colon cancer. Significantly high expression levels of Srx and Prxs were detected in the tumor tissues stained with anti-CD133 antibody compared to in adjacent normal tissues stained with anti-CD133 antibody (Fig. 2E, Supplementary Fig. S2).

To confirm the biological relevance of Srx-Prx expression in colon cancer, we finally analyzed the correlation between Srx and Prxs overexpression and colon cancer patient overall survival using the GENT2 dataset (Supplementary Fig. S3A – D). Especially, the Srx and Prx2 overexpression resulted in significant reduction of survival rate in colon cancer patients. The results suggested that the Srx-Prx redox system could maintain the stemness and survival of colon CSCs via elimination of ROS produced by the OXPHOS machinery in the mitochondria.

### 3.3 Human Srx maintains Prx protein stability and contributes to colon CSC stemness and survival

**Fig. 3:**
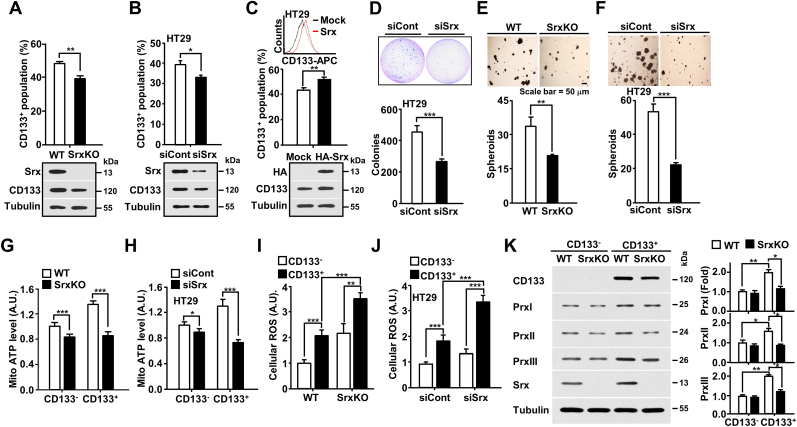
**Srx depletion-mediated collapse of the Srx-Prx redox system induces a significant decline in Prx expression and CSC population. (A**–**C)** CD133^+^ populations in Srx-depleted HT29-*Srx*KO (knockout) cells (A), *Srx*-targeting siRNA (si*Srx*)-transfected HT29 cells (B), and *Srx*-overexpressing HT29 cells (C) were analyzed using a FACSCanto flow cytometer. Protein levels detected by immunoblotting are shown in the bottom panel. **(D)** si*Srx*-transfected HT29 cells were subjected to a soft agar assay. The number of colonies generated per 10^4^ cells was counted after three weeks. **(E, F)** Srx-depleted HT29 cells (E) and si*Srx*-transfected HT29 cells (F) were subjected to sphere-formation assays in ultra-low-attachment 96-well plates. The number of spheroids generated per 10^4^ cells was counted after two weeks. Scale bar = 50 μm. **(G, H)** ATP levels were determined in Srx-depleted HT29-*Srx*KO cells (G) and si*Srx*-transfected HT29 cells (H) by quantifying the luciferase-catalyzed ATP-dependent oxidation of luciferin. **(I, J)** Srx-depleted cells (I) and si*Srx*-transfected HT29 cells (J) were sorted into CD133^+^ and CD133^–^ cells and intracellular ROS in sorted cells were measured using the CM-DCFDA dye. The fluorescence intensity is expressed in arbitrary units (A.U.) **(K)** HT29-*Srx*KO cells were sorted via magnetic-activated cell sorting into CD133^–^ and CD133^+^ populations. The cells were subjected to immunoblotting using the indicated antibodies. Western blot were carried out, and the intensities of PrxI, PrxII, and PrxIII proteins normalized to the loading control tubulin are presented. Statistical significance was defined as follows: *, *P* < 0.05; **, *P* < 0.01; and ***, *P* < 0.001. All the data in the study are expressed as the mean ± standard deviation obtained from the results of three independent experiments.

For the evaluation of *Srx* function in colon CSCs, we first constructed an Srx-depleted HT29 (HT29-*Srx*KO) colon cancer line using the CRISPR/Cas9 system (Supplementary Fig. S4A). Following treatment with *tert*-butyl hydroperoxide, we confirmed that the PrxI/II/III proteins were consistently hyperoxidized in the HT29-*Srx*KO cell line relative to wild-type (WT) HT29 cells (Supplementary Fig. S4B). Furthermore, using the HT29-*Srx*KO cell line and si*Srx* (siRNA against *Srx* gene) transfected cell lines, we identified a significant decline in the proportion of CSC population in HT29-*Srx*KO cells and si*Srx*-transfected HT29, HCT116, and SNUC5 cells relative to the respective control cell lines (Fig. 3A and B and Supplementary Figs. S4C and D). In contrast, *Srx* overexpression induced an increase in the number of CD133^+^ cells in HT29 cells (Fig. 3C). Next, to examine whether *Srx* modulated CSC-mediated tumorigenesis, we performed an anchorage-independent growth assay. All the si*Srx*-transfected cell lines exhibited significant reduction in colony formation compared to the control cell lines (Fig. 3D, Supplementary Figs. S4E and F). Moreover, sphere-formation assays revealed that sphere formation was reduced by approximately 40%–50% in HT29-*Srx*KO and si*Srx*-transfected cell lines (Fig. 3E and F and Supplementary Figs. S4G and H). Furthermore, mitochondrial ATP levels were markedly decreased in CSCs (CD133^+^ cells) in response to Srx depletion (Fig. 3G and H and Supplementary Figs. S4I and J). Thus, Srx depletion induced a decline in colon CSCs through multiple dysregulations in the tumorigenic ability of CSCs, stemness, and mitochondrial energy production, potentially by disrupting the Srx-Prx redox system. Next, to assess whether *Srx* depletion in colon CSCs led to the disruption of the Srx-Prx redox system, we measured changes in ROS levels and stability of Prx protein in Srx-depleted cells. Elevated ROS levels were detected in colon CSCs compared to in non-CSCs, and a substantial increase was noted in colon CSCs isolated from HT29-*Srx*KO and si*Srx*-transfected HT29 cells (Fig. 3I and J). The hyperoxidized-Prxs, which are generated by the absence of an electron recipient, are degraded by the proteolysis mechanism [19]. Hence, to assess whether changes in PrxI, PrxII, and PrxIII protein stability are caused by Srx depletion and increased ROS levels, we examined the half-life of Prx proteins in the presence of cycloheximide (CHX). Notably, Srx depletion resulted in a decline in PrxI, PrxII, and PrxIII protein stability (Supplementary Fig. S5A – D). Furthermore, declines in PrxI, PrxII, and PrxIII protein levels were noted in CSCs sorted from Srx-depleted HT29-*Srx*KO compared to in the control cells (Fig. 3K). Finally, we observed an increase in hyperoxidation of Prx in CSCs compared to in non-CSCs under reducing conditions using an alkylating agent, *N*-ethylmaleimide (NEM), to prevent artefactual oxidation of Prx during lysis and sample preparation. The high reactivity of Prxs with hydrogen peroxide facilitates dimerization upon cell lysis [24], whereas hyperoxidation does not occur during cell lysis, as the process is slow and requires catalytic cycling [25]. As shown in Supplementary Figs. S5E and 5F, in the CSCs in SrxKO cells and the short-term culture cells (2 weeks), after sorting according to RFP expression level in HT29-CD133-RFP cells, the monomerized Prxs (hyper-oxidized Prxs) are increased more than in non-CSCs and in the long-term culture cells (8 weeks). The hyperoxdiation of Prxs means that colon CSCs utilize the OXPHOS system to produce the ATP and generated higher amounts of ROS than non-CSCs, as by-products of the process. Overall, the results indicated that *Srx* maintained the stemness and tumorigenicity of colon CSCs via the reduction of hyperoxidized Prx proteins. Additionally, *Srx* aided the maintenance of peroxidase activity by regulating protein stability and hyperoxidation of Prxs proteins.

### 3.4 Srx depletion-mediated collapse of the Srx-Prx redox system results in DNA damage, cIAP1 destabilization, and mitochondrial dysfunction in colon CSCs

**Fig. 4:**
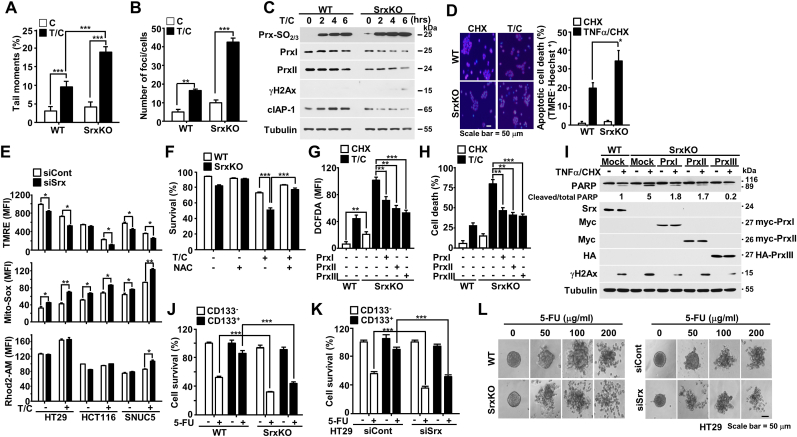
**Srx depletion sensitizes colon CSCs to death through multiple forms of damage.** (**A**) HT29-*Srx*KO cells were subjected to comet assays after TNFα/cycloheximide (CHX) treatment. Data in the graph represent the mean ± standard deviation of tail moments per cell from an average of 50–60 cells. (**B**) HT29-*Srx*KO cells were treated with TNFα/CHX. Foci were immunostained to detect γH2AX. The number of γH2AX foci per 50 cells was quantified. (**C**) Time-course treatment of HT29-*Srx*KO cells with TNFα/CHX and immunoblotting with the indicated antibodies. **(D)** TNFα/CHX-treated HT29-*Srx*KO cells were labeled with tetramethylrhodamine (TMRE) and Hoechst 33342. The shrunken shape of nuclei and depolarized mitochondria in the unstained cells by TMRE represent apoptotic cell death. Counts are presented in the right panel. **(E)** HT29, HCT116, and SNUC5 cells were transfected with si*Srx*, and mitochondrial activities, including membrane potential, mitochondrial ROS level, and mitochondrial calcium level, were measured. (**F**) HT29-*Srx*KO cells were pre-treated with *N*-acetylcysteine (NAC) for 1 h and exposed to TNFα/CHX treatment for 6 h. Cells were stained with Annexin V-FITC/PI and subjected to flow cytometry. (**G–I**) HT29-*Srx*KO cells were transduced with mock, PrxI, PrxII, or PrxIII-expressing lentiviral particles and treated with TNFα/CHX. Cells were subjected to ROS measurement as described in the Materials and Methods (G). Cells were stained with Annexin V-FITC/PI and analyzed using flow cytometry to confirm that Prx overexpression rescued cell death (H). Immunoblotting revealed a decline in PARP cleavage and the phosphorylation of H2AX, confirming the rescue of cell death by Prx overexpression (I). PARP cleavage was quantified by dividing the densitometric band intensity of the cleaved band by the total intensities of the full and cleaved band. (**J, K**) Sorted CD133^+^ and CD133^–^ cells from HT29-*Srx*KO (J) and si*Srx*-transfected HT29 cells (K) were treated with 5-fluorouracil (5-FU). Cells were stained with Annexin V-FITC/PI and analyzed using flow cytometry. **(L)** HT29-*Srx*KO cells and si*Srx*-transfected HT29 cells were plated in ultra-low-attachment plates. Spheroids were seeded (single spheroid per well) into 50 wells of ultra-low-attachment plates. Representative photomicrographs demonstrate the spheroid shape after 5-FU treatment. MFI, mean fluorescence intensity (E, G). Scale bar: 50 μm (D, L). Statistical significance was defined as follows: *, *P* < 0.05; **, *P* < 0.01; and ***, *P* < 0.001. All data in the study are expressed as the mean ± standard deviation obtained from three independent experiments.

To determine the cause of Srx depletion-mediated decline in stemness ability and survival of colon CSCs, we first investigated for signs of DNA damage due to the collapse of the Srx-Prx redox system. Using the Comet assay, an increase in DNA strand breakage by tumor necrosis factor-alpha/cycloheximide (TNFα/CHX) was observed in Srx-depleted cells (Fig. 4A, Supplementary Fig. S6A). Moreover, we showed the rescue of DNA damage by Srx depletion via experiments using Srx and PrxI-expressing lentiviruses (Supplementary Fig. S6B). Furthermore, we examined Srx-depleted cells for the formation of foci of phosphorylated H2AX (γH2Ax) at the DNA strand breaks, a hallmark of DNA damage response [26]. Immunofluorescence staining showed that *Srx* depletion notably increased the number of TNFα/CHX-induced γH2Ax foci in colon cancer cells (Fig. 4B, Supplementary Fig. S6C). Immunoblotting analysis also confirmed increased γH2Ax levels by Srx depletion in colon cancer cells (Fig. 4C, Supplementary Fig. S6D). Additionally, we observed a decline in PrxI, PrxII, and cIAP1 protein levels in Srx-depleted cells. Thus, our results indicated that Srx depletion led to the collapse of the Srx-Prx redox system with a decline in PrxI and PrxII protein expression, and subsequently increased DNA damage-induced cell death with a decline in cIAP1 expression. Moreover, to examine the potential involvement of mitochondria in cell death caused by Srx depletion, mitochondria and their chromosomes were co-stained using TMRE and Hoechst 33342 dyes. Notably, Srx-depleted colon cancer cells demonstrated distinct characteristics of apoptotic cell death, including reduced mitochondrial membrane potential and shrunken chromosome shapes (Fig. 4D, Supplementary Fig. S6E). We measured the membrane potential, mitochondrial ROS, and calcium production using TMRE, Mito-Sox, and Rhod2-AM dyes to confirm the alteration of mitochondrial function by Srx depletion (Fig. 4E). Compared to the control cells, Srx-depleted cells exhibited lower mitochondrial membrane potential and increased ROS levels, whereas no significant change in calcium level was observed.

To confirm whether the increased ROS levels and decreased Prx protein expression due to the collapse of the Srx-Prx redox system contributed to cell death, a rescue experiment was performed using PrxI, PrxII, and PrxIII-expressing lentiviruses and an *N*-acetylcysteine (NAC), a known ROS scavenger. As predicted, NAC treatment attenuated the increased cell death by TNFα/CHX in Srx-depleted cells (Fig. 4F). Similarly, transduction of cells with a Prx-expressing lentivirus effectively reduced the elevated levels of ROS induced by Srx depletion after TNFα/CHX treatment (Fig. 4G). Furthermore, flow cytometry cell death assays based on Annexin V-FITC/PI staining revealed that increased Prx (I, II, III) expression resulted in a decline in colon cancer cell death after Srx depletion and TNFα/CHX treatment (Fig. 4H). Using immunoblotting with anti-PARP antibody, we also confirmed enhanced cell survival after lentiviral-mediated Prx overexpression (Fig. 4I). Especially, as shown in Supplementary Fig. S6B and Fig. 4G–I, PrxI-overexpression rescued DNA damage (γH2AX) associated with the increased ROS, facilitating cell survival in Srx depleted cells. Therefore, overall, Srx depletion in colon cancer cells led to decreases in PrxI, PrxII, and PrxIII protein stability and mitochondria-mediated apoptotic cell death. Furthermore, cell death was triggered by an increase in DNA damage and a decrease in the expression of the anti-apoptotic protein cIAP, as well as mitochondrial dysfunction.

Finally, we assessed whether the collapse of the Srx-Prx redox system could sensitize colon CSCs to cell death in response to 5-FU, an anticancer drug. Indeed, Srx depletion sensitized the bulk tumor cells and CSCs to 5-FU-induced cell death by decreasing PrxI, PrxII, and PrxIII expression (Fig. 4J and K and Supplementary Figs. S6F and G). Moreover, the spheroids formed by Srx-depleted cells exhibited enhanced sensitivity to 5-FU treatment in a dose dependent manner when compared with spheroids formed from control cells (Fig. 4L). Overall, the results suggested that the Srx-Prx redox system eliminated ROS and was stabilized by Srx, thereby protecting colon CSCs from ROS produced in the mitochondrial OXPHOS.

### 3.5 Srx-Prx redox system is essential for tumor growth and metastasis by CSCs *in vivo*

**Fig. 5:**
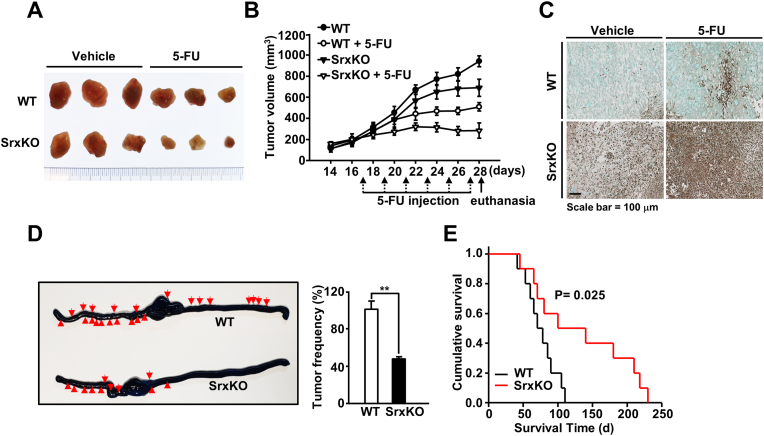
**Collapse of the Srx-Prx redox system suppresses tumor growth and metastasis in xenografts and ortho-xenografts in SCID mice.** The effect of Srx depletion on tumor growth kinetics was evaluated in four groups of mice (HT29-WT [wild-type], HT29-WT/5-FU, HT29-*Srx*KO, and HT29-*Srx*KO/5-FU). (**A**) Representative images of tumors harvested from mice in each group. (**B**) Data are presented as the mean tumor volumes recorded at two-day intervals ± standard error of the mean. (**C**) The tumor samples from each mouse were subjected to terminal deoxynucleotidyl transferase-mediated deoxyuridine triphosphate nick-end labeling staining. Scale bar = 100 μm. (**D**) Isolated CD133^+^ cells from HT29-WT or HT29-*Srx*KO cells were injected into the ceca of SCID mice to determine the effects on local invasion and metastasis in the colon. Ten weeks later, the colons were stained with methylene blue, and metastasis was quantified and presented as tumor frequency. Red arrows indicate the presence of a tumor. (**E**) Animal survival curves associated with WT and Srx-depleted cells. The cumulative survival values were plotted in relation to the time of observation post-injection, and significant differences between the two groups are shown. Statistical significance was defined as follows: **, *P* < 0.01. All the data in the study are expressed as the mean ± standard deviation obtained from the results of three independent experiments. (For interpretation of the references to colour in this figure legend, the reader is referred to the Web version of this article.)

We generated a mouse xenograft model to evaluate the anti-tumorigenic effects of a collapsed Srx-Prx redox system. CD133^+^ cells sorted from HT29-*Srx*KO or HT29-WT cells were injected into SCID mice, and the animals were monitored for tumor growth. The resultant tumors in the mice injected with Srx-depleted CD133^+^ cells were markedly smaller than those observed in mice injected with HT29-WT cells (Fig. 5A and B). Furthermore, Srx depletion resulted in an enhanced regression effect in 5-FU-treated mice compared to in the control mice (Fig. 5A and B). The effect was due to the increased apoptosis observed in HT29-*Srx*KO and 5-FU-treated tumors, as shown by terminal deoxynucleotidyl transferase-mediated deoxyuridine triphosphate nick-end labeling staining (Fig. 5C). Subsequently, to examine the invasion and metastasis of cells, we used an orthotopic xenograft system and performed transplantation of the tumor generated from CD133^+^ cells of HT29-*Srx*KO and HT29-WT into the ceca of SCID mice. The colon CSCs in both the groups of mice were locally invasive and caused colon cancer metastasis. However, the frequency of local invasion and metastasis decreased in mice with cecum-injected Srx-depleted CSCs (Fig. 5D). The mice that received Srx-depleted cells exhibited an improved prognosis compared to those that received WT control cells (Fig. 5E). The improvement was likely due to the decreased tumor frequency observed in the Srx-depleted group (Fig. 5D and E). Collectively, the *in vivo* results implied that as a component of the Srx-Prx redox system, Srx functioned to eliminate ROS, which are side-products of energy metabolism, and contributed to anticancer drug resistance, increased stem cell-like properties, and distal metastasis in colon CSCs. Thus, Srx can be considered a pivotal survival factor, Prxs regulator, and potential therapeutic target for treating colon cancer.

### 3.6 Combined treatment with 5-FU and frugoside induces colon CSC death via the collapse of the Srx-Prx redox system

**Fig. 6:**
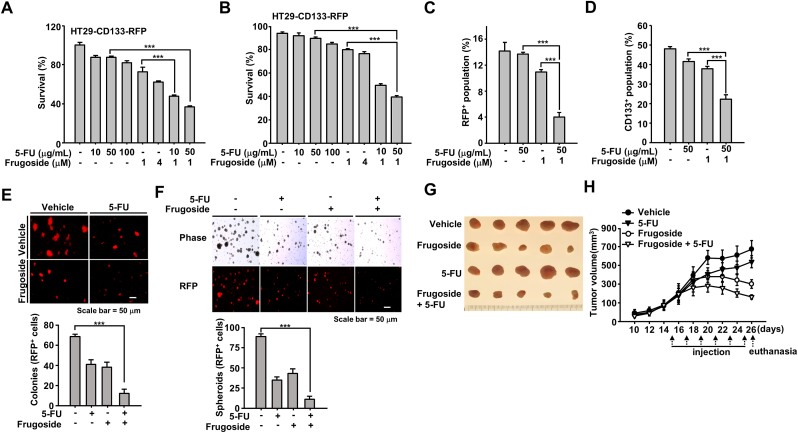
**Combined exposure to the Srx inhibitor, frugoside, and 5-FU sensitizes CSCs to cell death.** (**A, B**) HT29-CD133-RFP cells were co-treated with 5-FU and frugoside at the indicated doses for 24 h. The cells were subjected to a CCK-8 assay (A) and flow cytometric analysis after staining with Annexin V-FITC/PI (B). (**C, D**) HT29-CD133-RFP cells were co-treated with 5-FU and frugoside at the indicated doses for 24 h. CD133^+^ populations exhibiting red fluorescence (C) and stained with an anti-CD133 (D) antibody were analyzed using flow cytometry. (**E, F**) HT29-CD133-RFP cells were subjected to soft agar assays (E) and sphere-formation assays (F) in a medium containing 5-FU (50 μg/ml)/frugoside (1 μM). The number of colonies generated per 10^4^ cells was counted three weeks later using a fluorescence microscope. Spheroids were generated in low attachment plates and counted two weeks later. Scale bar = 50 μm. (**G-H**) The effect of Srx inhibitor on the tumor growth kinetics was evaluated in four groups of mice (HT29, HT29-5-FU, HT29-frugoside, and HT29-5-FU/frugoside). (G) Representative images of tumors harvested from mice in each group. (H) Data are presented as the mean tumor volumes recorded at two-day intervals ± standard error of the mean. Statistical significance was defined as follows: ***, *P* < 0.001. All the data in the study are expressed as the mean ± standard deviation obtained from the results of three independent experiments. (For interpretation of the references to colour in this figure legend, the reader is referred to the Web version of this article.)

Frugoside, an Srx-targeting drug, suppresses Srx and induces mitochondrial-mediated apoptotic cell death [20]. Hence, in this study, we assessed whether the collapse of the Srx-Prx redox system following treatment with frugoside in combination with 5-FU could contribute to cell death. Using the CCK-8 assays, we observed that the combined treatment induced a 30% increase in cell death compared to those in a single treatment with 5-FU or frugoside in HT29-CD133-RFP (Fig. 6A). In addition, in the Annexin V-FITC/PI staining, there was a significant increase in HT29-CD133-RFP cell death after the combined treatment with 5-FU and frugoside (Fig. 6B). To validate that the combination treatment specifically reduced the CD133^+^ population in the HT29-CD133-RFP cell line, we assessed CD133^+^ cells via FACS analysis for RFP fluorescence (Fig. 6C) or after labeling the cells with anti-CD133-allophycocyanin (APC) antibody (Fig. 6D). In both the assessments, the CD133^+^ cell population decreased in response to the combination treatment and exhibited similar alteration patterns (Fig. 6C and D).

We also evaluated the effect of the combined treatment on the tumorigenicity of CSCs. Using the colony (Fig. 6E) and sphere-formation assays (Fig. 6F), we demonstrated that the resultant colonies and spheroids were significantly smaller in the 5FU-frugoside-treated HT29 cell group, which included the CD133^+^-RFP^+^-CSC population, than in groups treated with 5-FU or frugoside alone. Finally, we evaluated the potential biological application of combined5-FU and frugoside treatment using an animal model. As shown in Fig. 6G and H, the resultant tumors in the mice treated with 5-FU or frugoside were smaller than those observed in control mice. Furthermore, combined 5-FU and frugoside treatment resulted in markedly enhanced regression effects in mice treated separately with 5-FU or frugoside. Collectively, the results highlight the potency, safety, and cancer cell specificity of a potential pharmacological approach for targeting Srx in colon CSCs. Furthermore, frugoside is a potential adjunctive therapeutic agent that selectively inhibits Srx in colon cancer cells.

### 3.7 Metabolic adaptation of colon CSCs is mediated by transcriptional regulators *Nrf2* and *FoxM1*

**Fig. 7:**
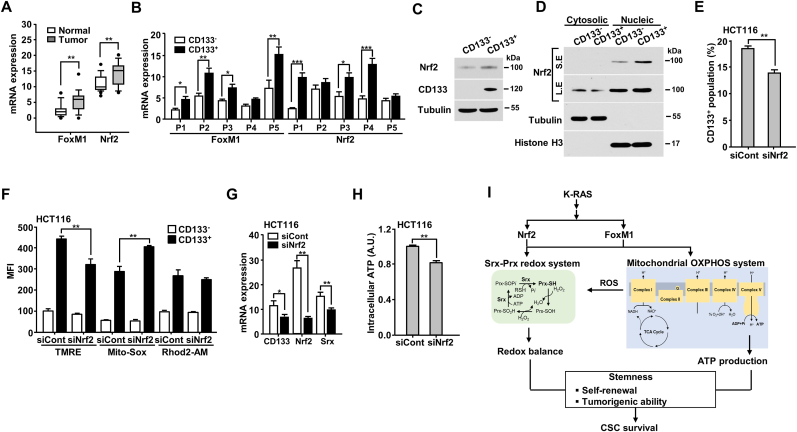
**Nrf2 and FoxM1 transcription activates Srx and Prxs in colon cancer cells.** (**A**) The transcriptional levels of *Nrf2* and *FoxM1* in cells isolated from colon cancer patients were analyzed using a quantitative real-time polymerase chain reaction (qRT-PCR). (**B**) The transcriptional levels of *Nrf2* and *FoxM1* in CSCs and non-CSCs isolated from colon cancer patients were analyzed using qRT-PCR. (**C**) HCT116 cells were sorted into CD133^-^ and CD133^+^ populations and then subjected to immunoblotting with indicated antibodies. (**D**) CD133^-^ and CD133^+^ cells isolated from HCT116 cells were fractionated into cytosolic and nucleic fractions, and then the fractions were analyzed by immunoblotting with indicated antibodies. Short exposure (S.E) and long exposure (L.E) (**E**) The CD133^+^ population was analyzed using a flow cytometer in si*Nrf2*-transfected HCT116 cells. (**F**) The mitochondrial membrane potential (TMRE), ROS levels (Mito-Sox), and calcium levels (Rhod2-AM) were measured in sorted CD133^+^ cells from HCT116 colon cancer cells. MFI, mean fluorescence intensity. (**G**) The decrease in *CD133* and *Srx* expression by *Nrf2* depletion was confirmed using qPCR in HCT116 cells. (**H**) The ATP levels were measured by quantifying the luciferase-catalyzed ATP-dependent oxidation of luciferin in *Nrf2*-depleted and control cells. (**I**) Schematic illustration of the expression and function of Srx-Prx redox system, induced by *Nrf2* and *FoxM1* in colon CSCs. In CSCs, Srx-Prxs is significantly upregulated via the activation of *Nrf2* and *FoxM1* transcription factor. Elevated Srx-Prxs maintain the redox balance and contribute to ATP production by the mitochondrial OXPHOS in colon CSCs. *Nrf2* and *FoxM1* also induce mitochondrial biogenesis, which is critical for maintaining the self-renewal and tumorigenic abilities of colon CSCs. Statistical significance was defined as follows: *, *P* < 0.05; **, *P* < 0.01; and ***, *P* < 0.001. All the data in the study are expressed as the mean ± standard deviation obtained from the results of three independent experiments.

Nuclear factor erythroid2-related factor 2 (*Nrf2*) and forkhead box protein M1 (*FoxM1*) are essential regulators for protecting cancerous cells from oxidative stress caused by the mitochondrial OXPHOS system [[27], [28], [29]]. Nrf2 is a well-known transcriptional regulator of *Srx, PrxI,* and *PrxII* genes [30,31]. *FoxM1* enhances mitochondrial function to maintain stemness and survival of CSCs by acting as a regulator of *PrxIII* [6]. Hence, considering their roles in cancerous cells and CSCs, we first verified *Nrf2* and *FoxM1* expression in colon cancer tissues and adjacent normal tissues using qRT-PCR. *Nrf2* and *FoxM1* expression was increased in colon cancer tissues compared to in adjacent normal tissues (Fig. 7A). In addition, *Nrf2* and *FoxM1* expression was increased in CSCs isolated from colon cancer tissues compared to in non-CSCs (Fig. 7B). Especially, we showed that the levels of Nrf2 expression were higher in CSCs than in non-CSCs (Fig. 7C), and the Nrf2 protein translocation into nuclei as transcriptional factor was much more in CSCs than in non-CSCs (Fig. 7D). To determine the role of *Nrf2* in CSC, we suppressed *Nrf2* expression using an siRNA approach and then isolated CSCs. We observed a significant decline in CSC population in si*Nrf2*-transfected cells compared to in siControl-transfected CSCs. The decline corresponded to a significant increase of superoxide (O_2_^•−^) in mitochondria (Mito-Sox) and decreased mitochondrial membrane potential (TMRE; Fig. 7E and F). We observed no change in Ca^2+^ levels (Rhod2-AM) in the assessed groups. Additionally, we observed lower CD133 and Srx expression levels in si*Nrf2*-transfected cells than in the control cells (Fig. 7G). ATP levels were also markedly decreased in the mitochondria of si*Nrf2*-transfected cells (Fig. 7H). Collectively, the findings of this study and prior studies demonstrate that *Nrf2* and *FoxM1* enhance the mitochondrial OXPHOS system and upregulate Srx and Prxs to maintain the stemness and survival of CSCs via the elimination of ROS, a by-product of OXPHOS.

## 4 Discussion

In the present study, we uncovered the need for coordination of the Srx-Prx redox system and the mitochondrial OXPHOS to maintain the stemness and survival of colon CSCs. We further demonstrated that Srx from the Srx-Prx redox system contributed to the stability of PrxI, PrxII, and PrxIII proteins as a master enzyme to catalyze the reduction of hyperoxidized Prxs in colon CSCs. Thus, increased Srx expression played a critical role in colon cancer and was important for promoting the stemness and survival of colon CSCs and maintaining the mitochondrial OXPHOS system via the elimination of ROS produced during ATP production in CSCs.

The metabolic states of CSCs have not been thoroughly investigated. Recently, several studies have reported the energy metabolism features of CSCs. Pancreatic CSCs have been shown to use galactose as a carbon source to utilize the mitochondrial OXPHOS system to produce ATP [9]. Colon CSCs also utilize the primary mitochondrial OXPHOS system rather than glycolysis [6]. Additionally, targeting the mitochondrial bioenergetics induces cell death in ovarian CSCs [32]. Consistent with these studies, we elucidated the energy metabolism-related properties of colon CSCs. Notably, using RFP-labeled-CSCs, we demonstrated an alteration of energy metabolism from CSCs to bulk tumor cells via metabolic reprogramming (Fig. 1, Supplementary Fig. S1). Furthermore, we showed that CSCs, isolated from the bulk tumor, preferentially utilized the mitochondrial OXPHOS system, regardless of the metabolic system in the bulk tumor. Such metabolic properties of CSCs may be required to maintain the stemness ability and differentiate bulk tumor cells. Therefore, an enhanced understanding the metabolic properties of colon CSCs could facilitate the development of enhanced CSC-specific therapeutic targets.

Prxs play the common function of H_2_O_2_ scavenging, and furthermore, they are not simply redundant proteins but play critical roles in cancer development and progression [33,34]. Several studies have reported that PrxI loss results in increased oxidative DNA damage in cancer cells [[35], [36], [37]]. PrxII also regulates apoptosis by stabilizing cIAP1 [37], while PrxIII helps in the maintenance of mitochondrial energy metabolism [6]. To determine whether Srx protects cancer cells via the maintenance of the peroxidase activities of PrxI, PrxII, and PrxIII in response to apoptotic stimuli, we depleted Srx in colon cancer cells. We found that Srx depletion resulted in decreased PrxI, PrxII, and PrxIII levels and increased mitochondria-mediated apoptotic cell death due to increased DNA damage, decreased anti-apoptotic cIAP expression, and mitochondrial dysfunction (Fig. 3, Fig. 4 and Supplementary Figs. S5 and 6). Furthermore, the collapse of the Srx-Prx redox system by Srx depletion significantly reduced the level of ATP in CSCs along with a marginal decrease in non-CSCs. The rescue experiment involving Prx overexpression in Srx-depleted cells confirmed that Srx tightly regulated the functions of Prxs via the Srx–Prx axis (Fig. 4, Supplementary Fig. S6).

Frugoside is an Srx inhibitor that suppresses Srx expression and induces mitochondria-mediated apoptotic cell death in melanoma cells [20]. In the present study, we demonstrated that combined treatment with frugoside and 5-FU inhibited colon CSCs and reduced the tumorigenicity of the cells (Fig. 6). The data provide compelling evidence of the potential use of Srx as a target in colon cancer treatment. Furthermore, the significant effect of combined frugoside and 5-FU treatment in colon CSCs indicates that such a strategy may be a more effective anticancer adjunct combination therapy.

Recent evidence has shown that cancer tissues are more sensitive to oxidative stress because they generate higher levels of ROS when compared to normal cells. Therefore, an increase in intracellular ROS concentrations or the collapse of the cellular redox balance could selectively kill tumor cells without causing severe damage to normal tissues [[38], [39], [40]]. However, the development of antioxidant protein-targeted drugs that can induce ROS overproduction in cancer cells has been complex owing to the existence of several ROS-regulating systems. These include the Prx–Trx–TrxR–Srx axis, glutathione/glutathione peroxidase, OXPHOS, superoxide dismutase, and catalase, in addition to their complementary actions that eliminate the excess ROS in cells [[41], [42], [43], [44]]. However, if antioxidant inhibitors can induce ROS overproduction via the simultaneous regulation of several antioxidants, they may be helpful for the treatment of incurable cancers via elimination of CSCs. Consistently, our data suggested that Srx acted as a multi-regulator by reducing the hyperoxidized PrxI, PrxII, and PrxIII proteins, and thus is a prospective therapeutic target against incurable colon cancer.

Our study also demonstrated that the energy metabolism and redox system of colon CSCs must be tightly regulated to maintain their stemness. Increased ROS levels can inhibit self-renewal in stem cells by promoting cell differentiation and senescence/apoptosis induction in both normal stem cells and colon CSCs [45,46]; therefore, colon CSCs must eliminate ROS produced via the OXPHOS system. Thus, the metabolic reprogramming of colon CSCs may involve the coordinated action of energy metabolism and redox systems.

Several studies have implicated *Nrf2* and *FoxM1* in tumorigenesis, as they are upregulated in various types of cancers [29,47] and are dependent on oncogenic signaling downstream of Ras activation [48,49]. In the present study, we reported that the transcription factors *Nrf2* and *FoxM1* are regulators of Srx and Prxs of the Srx-Prx redox system (Fig. 7). *Nrf2* and *FoxM1* expression was upregulated in colon tumor tissues compared to in the adjacent normal tissues. Moreover, *Nrf2* and *FoxM1* were highly expressed in colon CSCs compared to in non-CSCs isolated from colon cancer patients. Thus, our data indicated that *Nrf2* and *FoxM1* are key regulators of colon CSCs and are potentially attractive anticancer therapeutic targets. Furthermore, upregulated *Nrf2* and *FoxM1* expression during the metabolic alteration of colon CSCs may lead to the activation of the mitochondrial OXPHOS system and increase Srx and Prx expression in the Srx-Prx redox system.

Overall, to the best of our knowledge, this is the first study to report that the Srx–Prx redox system acts as a regulator of the mitochondrial OXPHOS in colon CSCs. The upregulation of Srx and Prx expression and the OXPHOS system by *Nrf2*/*FoxM1* promotes survival and stemness in colon CSCs. In addition, upregulated Srx expression facilitates the maintenance of the mitochondrial OXPHOS system through ROS elimination by Prx protein stabilization (Fig. 7I). Finally, we reported that Srx depletion sensitized colon CSCs to anticancer drug-induced cell death. Therefore, we successfully demonstrated that a combination treatment approach involving the use of Srx–Prx redox system-targeting compounds, such as frugoside, and anticancer drugs, is a promising and novel therapeutic strategy for colon cancer treatment.

## Funding

This study was supported by a grant (2021IL0029-1) from the 10.13039/501100005006Asan Institute for Life Sciences, Asan Medical Center, Seoul, Korea. This study was also supported by a 10.13039/501100003725National Research Foundation of Korea grant, funded by the Ministry of Science and ICT (No. 2019R1A2C1090362), and the Basic Science Research Program of the 10.13039/501100003725National Research Foundation of Korea, funded by the Ministry of Education (No. 2018R1D1A1B07044392, 2020R1A4A1016029). This work was also supported by the Technology Innovation Program, funded by the Ministry of Trade, Industry & Energy (MI, Korea) (20009707). The sponsors had no role in study design; in the collection, analysis and interpretation of data; in the writing of the report; and in the decision to submit the article for publication.

## Author contributions

I.-S.S. and S.-W.J. designed the research; I.-S.S., Y.J.J., Y.J., Y.-H.P., S.J.K., D.-W.E, S.-M.H., S.S., S.-U.K., and S.-W.J. performed the experiments; I.-S. S., Y.J.J., Y.J., Y.-H.P., S.J.K., D.-W.E, S.-M.H., P.C. L., S.-U.K., and S.-W.J. analyzed the data; and I.-S.S. and S.-W.J. wrote the manuscript.

## Data availability

The data that support the findings of this study are available from the corresponding authors upon reasonable request. Source data are provided with this paper.

## Ethics declarations

Human tissues were obtained in accordance with the ethical standards of the Institutional Review Board for Human Research at Inje University Busan Paik Hospital (approval number: 15–0081). All institutional and national guidelines for the care and use of laboratory animals were followed (No.2018-12-217). All animal procedures were approved by the Institutional Animal Care and Use Committee of the Asan Institute for Life Sciences (Republic of Korea).

## Declaration of competing interest

The authors declare no competing interests.

## Appendix A Supplementary data

Multimedia component 3 Multimedia component 3

figs1 figs1

figs2 figs2

figs3 figs3

figs4 figs4

figs5 figs5

figs6 figs6
