# Supplementary material for: The sulfiredoxin-peroxiredoxin redox system regulates the stemness and survival of colon cancer stem cells
Source: Redox Biol. 2021 Nov 15;48:102190. doi: 10.1016/j.redox.2021.102190 (PMC8605387; doi:10.1016/j.redox.2021.102190)
Supplement: Multimedia component 1 [file mmc1.docx]

**Supplementary Information**

**Supplementary Figure Legends**

**Figure S1. Tagging endogenous human CD133 with TagRFP in HT29 cells.** (**A**) Design of the targeting vector and sgRNA for generating knock-in (KI) CD133-RFP reporter. The human CD133 genomic sequence was tagged by two copies (2X) of TagRFP linked by the 2A sequence. The endogenous *CD133* gene was intact, and both CD133 and TagRFP were driven by the endogenous *CD133* promoter. The predicted Cas9-gRNA cutting position has been indicated with a sunder bolt symbol. The sequence including GGG PAM targeted by gRNA has been highlighted by a green underline. (**B**) Junction PCR analyses confirmed the site-specific targeting in the genome of HT29 cells. The PCR analysis was designed to specifically amplify the TagRFP insert. When the TagRFP reporter was successfully inserted in at least one endogenous *CD133* allele, it resulted in a fragment size of 1935 bp. Sanger sequencing confirmed the precise KI of the exogenous reporter. (**C**) Two representative images demonstrated the RFP expression in transfected HT29 cells maintained for seven days (magnification, 200 ×) and 14 days (magnification, 200 ×) after puromycin selection, respectively. The cells expressing RFP were characterized by sphere-forming capacity. Scale bar = 100 µm.

**Figure S2.** **Increased expression of** **Srx, PrxI, PrxII, and PrxIII in CSCs of colon cancer tissue.** The expression of Srx, PrxI, PrxII, PrxIII, and CD133 was observed in colon cancer tissues and adjacent normal tissues using immunohistochemistry. Red arrows are indicative of the cells with high expression of the assessed protein. Scale bar = 100 µm.

**Figure S3. Survival analysis of CRC using the GENT2 dataset.** Survival curves representing the survival of patients with colon cancer with high (red) and low (black) SRXN1 (**A**), PRDX1 (**B**), PRDX2 (**C**), and PRDX3 (**D**) expression. High expression was significantly (p<0.05) positively associated with poor OS according to the Kaplan-Meier (KM) plotter web tool (using GENT2)

**Figure S4. The Srx-Prx redox system maintains the stemness and survival of colon CSCs.** (**A**) A schematic illustration of *Srx* gene editing to reduce *Srx* expression using a CRISPR/Cas9. (**B**) For validating the established Srx-depleted HT29 cell line, the cells were treated for 10 min with 100 μM tert-butyl hydroperoxide (tBHP). After 10 min, tBHP was washed off, and the cells were incubated for the indicated durations with fresh medium. As a control, symbol C was not stimulated with tBHP. The lysates were assessed using immunoblotting with antibodies against the indicated proteins. (**C, D**) The CD133^+^ populations were analyzed using a FACSCanto flow cytometer in si*Srx*-transfected HCT116 (C) and SNUC5 cells (D). (**E, F**) si*Srx*-transfected HCT116 (E) and SNUC5 cells (F) were subjected to soft agar assay. The number of colonies generated per 10^4^ cells was counted three weeks later. (**G, H**) Srx-depleted HCT116 (G) and SNUC5 cells (H) were subjected to the sphere formation assay on ultra-low-attachment 96-well plates. The number of spheroids generated per 10^4^ cells was counted two weeks later. Scale bar = 50 µm. (**I, J**) The ATP levels were determined by quantifying the luciferase-catalyzed ATP-dependent oxidation of luciferin in Srx-depleted HCT116 (I) and SNUC5 cells (J). Statistical significance was defined as follows: *, *P* < 0.05; **, *P* < 0.01. All the data in the study are expressed as the mean ± standard deviation obtained from the results of three independent experiments.

**Figure S5**. **Collapse of the Srx-Prx redox system by Srx depletion results in decreased expression of Prx proteins by hyperoxidation, leading to ROS elevation.** (**A, B**) To evaluate the effect of Srx depletion in the stability of Prx proteins, HT29-*Srx*KO cells were pretreated with cycloheximide (CHX) and treated with tBHP for the indicated durations. The cells were then subjected to immunoblotting with anti-PrxI, -PrxII, and -PrxIII antibodies. Densitometry was performed and has been represented in the graph (B). (**C, D**) HT29 cells were transfected with siRNA against the *Srx* gene; then, the cells were treated with tBHP for the indicated durations after a CHX pretreatment. The cells were then subjected to immunoblotting (C). The resulting band intensity was measured and is represented in the graph (D). (**E, F**) Prx hyperoxidation was monitored by immunoblotting in non-reducing condition. HT29-WT and SrxKO cells (E) or the 2-week short-term culture cells and 8-week long-term culture cells (F) were incubated in 50 mM NEM for 10 min and lysed in the presence of NEM. Lysates were separated using non-reducing PAGE and immunoblotted using indicated antibodies.

**Figure S6.** **Disruption of** **the** **Srx-Prx redox system by Srx depletion sensitizes colon CSCs to cell death.** (**A**) si*Srx*-transfected HT29 cells were subjected to the Comet assay following TNFα/CHX treatment. Data show the mean ± SD of the tail moments per cell (average, 50–60 cells). (**B**) si*Srx*-transfected HT29 cells were treated with TNFα/CHX, and the resulting foci were immunostained with an anti-γH2AX antibody. (**C**) si*Srx*-transfected HT29 cells were treated with TNFα/CHX for the indicated durations and subjected to immunoblotting with the indicated antibodies. (**D**) si*Srx*-transfected HT29 cells were labeled with TMRE and Hoechst 33342 dye after treatment with TNFα/CHX. The graph represents the mean percentage of cells undergoing apoptotic cell death. (**E, F**) Sorted CD133^+^ cells and CD133^–^ cells from si*Srx*-transfected HCT116 (E) and SNUC5 cells (F) were treated with 100 μg/mL 5-FU. Cell death was analyzed using a FACSCanto flow cytometer after staining with Annexin V-FITC/PI. Scale bar = 50 µm (A, D).
