# Supplementary material for: The sulfiredoxin-peroxiredoxin redox system regulates the stemness and survival of colon cancer stem cells
Source: Redox Biol. 2021 Nov 15;48:102190. doi: 10.1016/j.redox.2021.102190 (PMC8605387; doi:10.1016/j.redox.2021.102190)
Supplement: Multimedia component 2 [file mmc2.docx]

**Supplementary Table 1. qRT-PCR primer sequences used**

| Gene | Accession  number | Primer sequences | | Product size (bp) |
| --- | --- | --- | --- | --- |
|  |  | Forward | Reverse |  |
| Srx | NM_080725.1 | CATCGATGTCCTCTGGATCA | CTGCAAGTCTGGTGTGGATG | 178 |
| PrxI | NM_181696.1 | GGGTATTCTTCGGCAGATCA | GCAGCCTGGCACTAAAACAG | 221 |
| PrxII | NM_005809.5 | GTGTCCTTCGCCAGATCACT | ACGTTGGGCTTAATCGTGTC | 154 |
| PrxIII | NM_006793 | GTTGTCGCAGTCTCAGTGGA | GACGCTCAAATGCTTGATGA | 216 |
| CD133 | NM_006017 | GCAATCTCCCTGTTGGTGAT | TCAGATCTGTGAACGCCTTG | 217 |
| CD44 | NM_000610.3 | TCTGTGCAGCAAACAACACA | TAGGGTTGCTGGGGTAGATG | 234 |
| Trx1 | NM_003329 | GTAGTTGACTTCTCAGCCACGTG | CTGACAGTCATCCACATCTACTTC | 120 |
| Trx2 | NM_012473.4 | GGACCTGACTTTCAAGACCGAG | GCCACCATCTTCTCTAACCTCG | 116 |
| UQCRC1 | NM_003365.2 | GGGCAAAAACATCCTCAGAA | ACGGATCCGGTTGTAGTCTG | 247 |
| SOD1 | NM_000454.4 | AGGGCATCATCAATTTCGAG | ACATTGCCCAAGTCTCCAAC | 217 |
| SOD2 | NM_000636.2 | GGAAGCCATCAAACGTGACT | ACACATCAATCCCCAGCAGT | 200 |
| TFAM | NM_003201.1 | CCGAGGTGGTTTTCATCTGT | TCCGCCCTATAAGCATCTTG | 203 |
| HO-1 | NM_002133 | CCAGGCAGAGAATGCTGAGTTC | AAGACTGGGCTCTCCTTGTTGC | 144 |
| GAPDH | NM_002046.7 | ctctgctcctcctgttcgac | aatccgttgactccgacctt | 105 |
