# Supplementary material for: The sulfiredoxin-peroxiredoxin redox system regulates the stemness and survival of colon cancer stem cells
Source: Redox Biol. 2021 Nov 15;48:102190. doi: 10.1016/j.redox.2021.102190 (PMC8605387; doi:10.1016/j.redox.2021.102190)
Supplement: Multimedia component 3 [file mmc3.docx]

Supplementary Table2. Case description and tumor features

| **Patients** | **Age/Sex** | **Site** | **Dukes** | **MSI** | **TNM** | **Tumor size** | **CD133 %** |
| --- | --- | --- | --- | --- | --- | --- | --- |
| P1 | 72/M | Rectosigmoid colon | C | low | T3N1a | 3.8*3.5cm | 1.5 |
| P2 | 30/M | Rectosigmoid colon | C | Stable (MSS) | T3N1b | 4*4cm | 1.4 |
| P3 | 53/M | Sigmoid colon | A | Stable (MSS) | T1N0 | 1.7*1.1cm | 0.8 |
| P4 | 53/M | Hepatic flexure | C | High | T3N1 | 5.5*4.5cm | 2.4 |
| P5 | 55/F | Rectosigmoid colon | C | High | T3N2a | 4*4cm | 1.2 |
| P6 | 63/F | Cecum | B | High | T3N0 | 4.0*3.3cm | 1.2 |
