# Supplementary figures and images for: The sulfiredoxin-peroxiredoxin redox system regulates the stemness and survival of colon cancer stem cells
Source: Redox Biol. 2021 Nov 15;48:102190. doi: 10.1016/j.redox.2021.102190 (PMC8605387; doi:10.1016/j.redox.2021.102190)

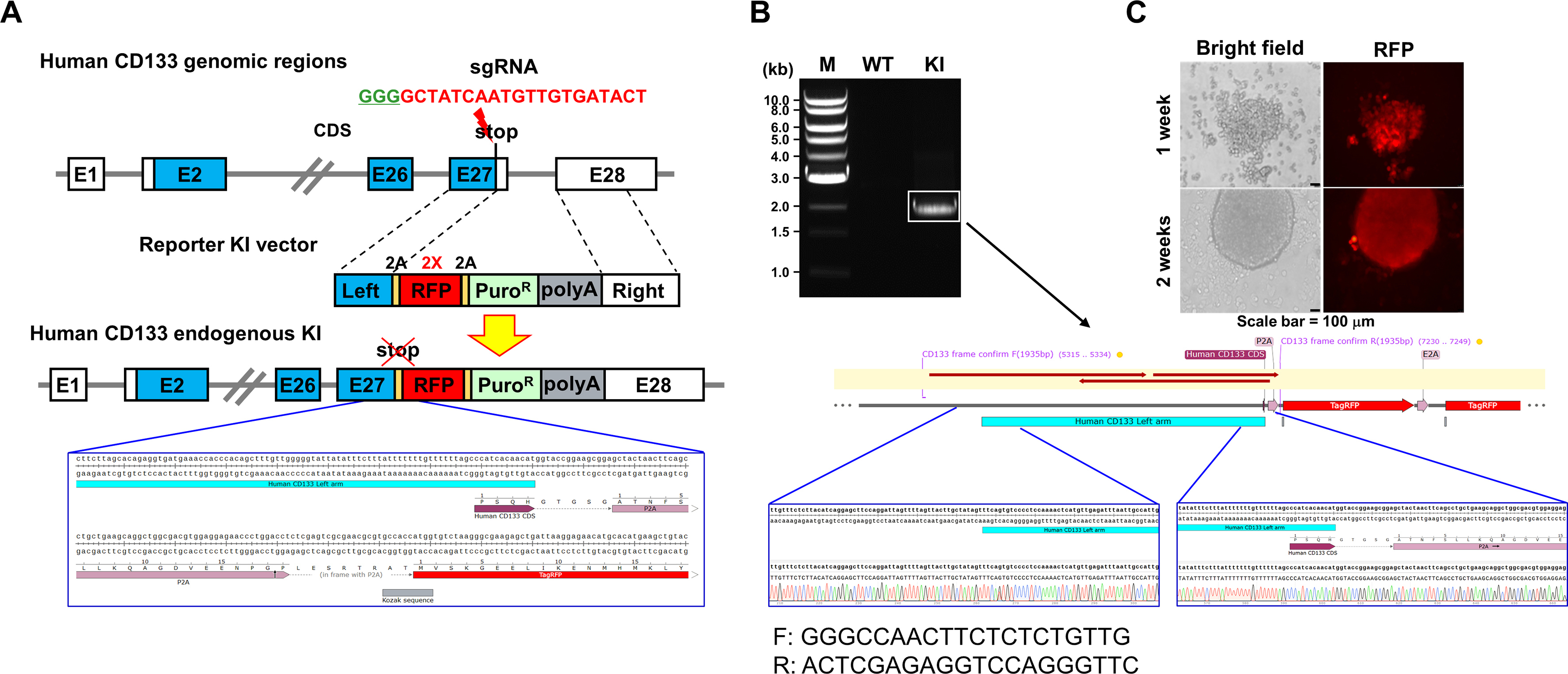

Supplement: figs1 [file mmcfigs1.jpg]

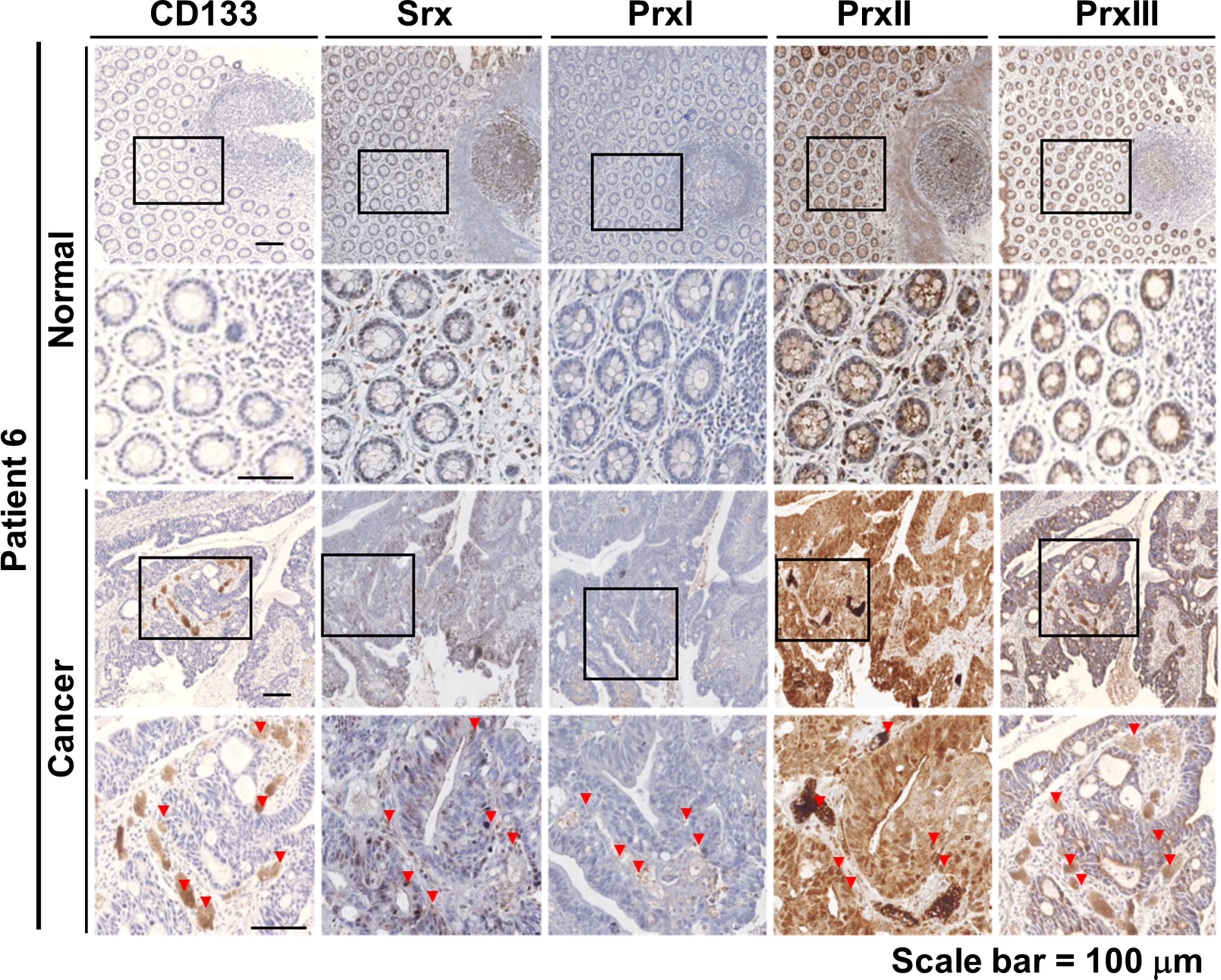

Supplement: figs2 [file mmcfigs2.jpg]

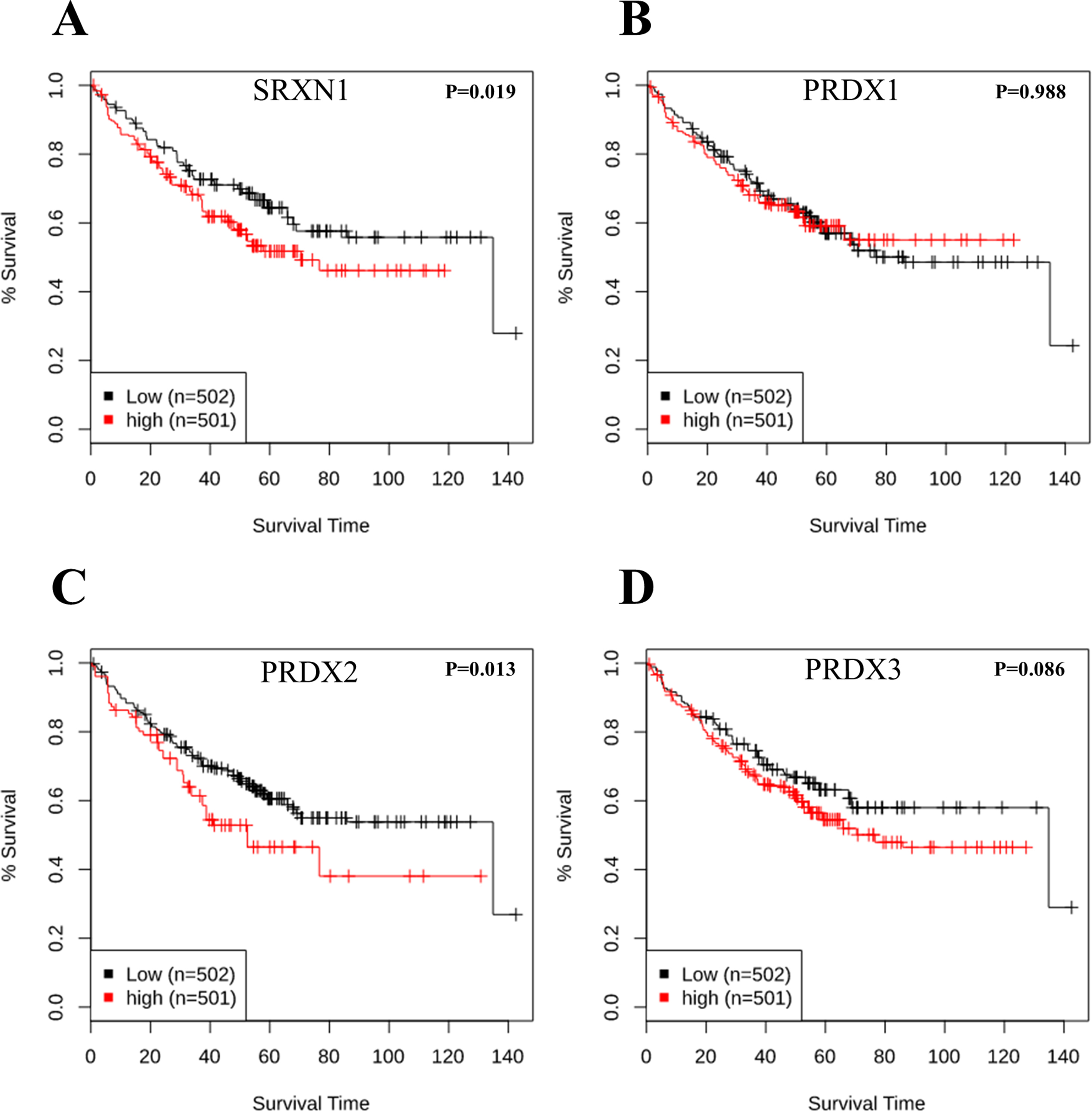

Supplement: figs3 [file mmcfigs3.jpg]

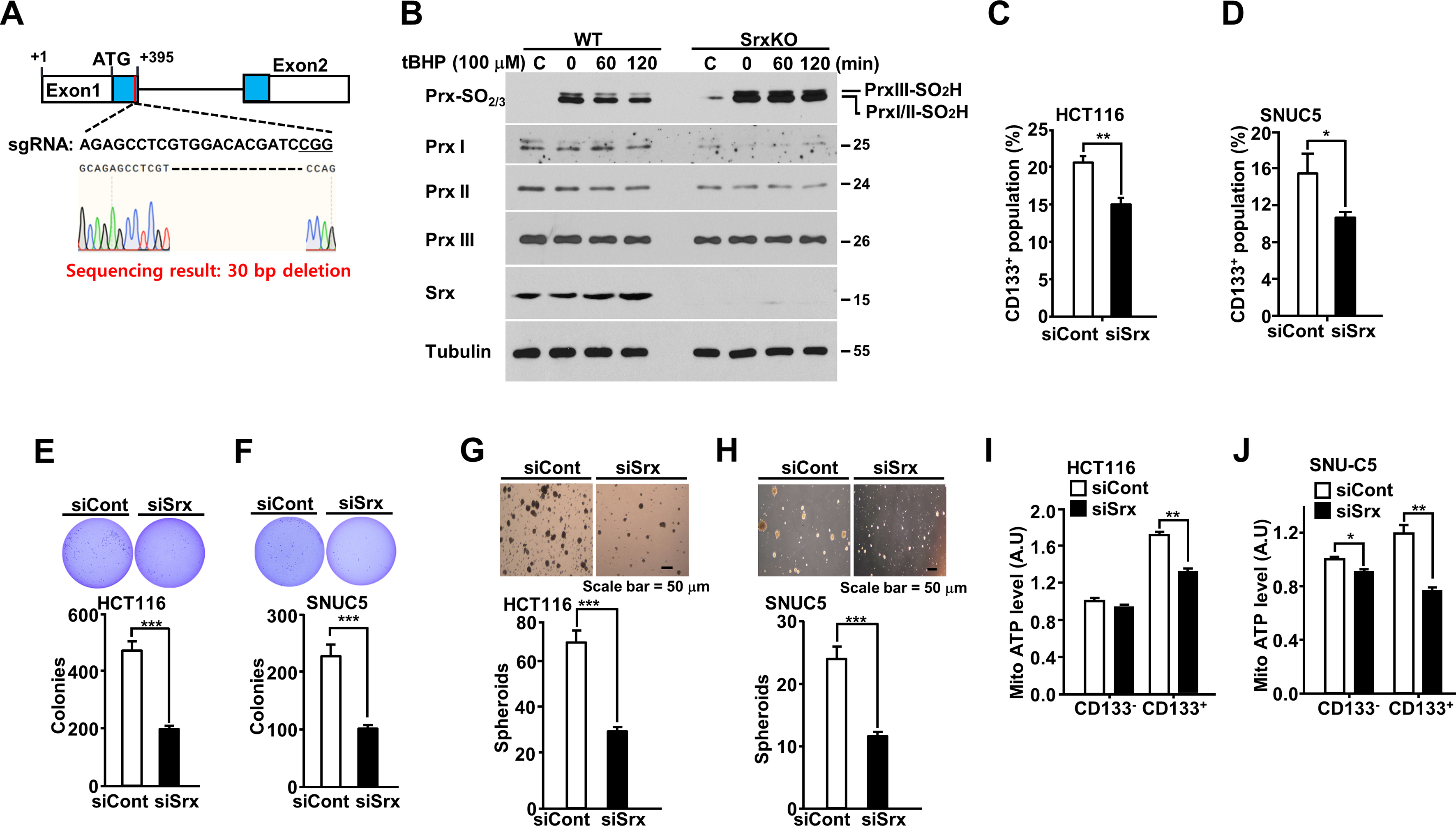

Supplement: figs4 [file mmcfigs4.jpg]

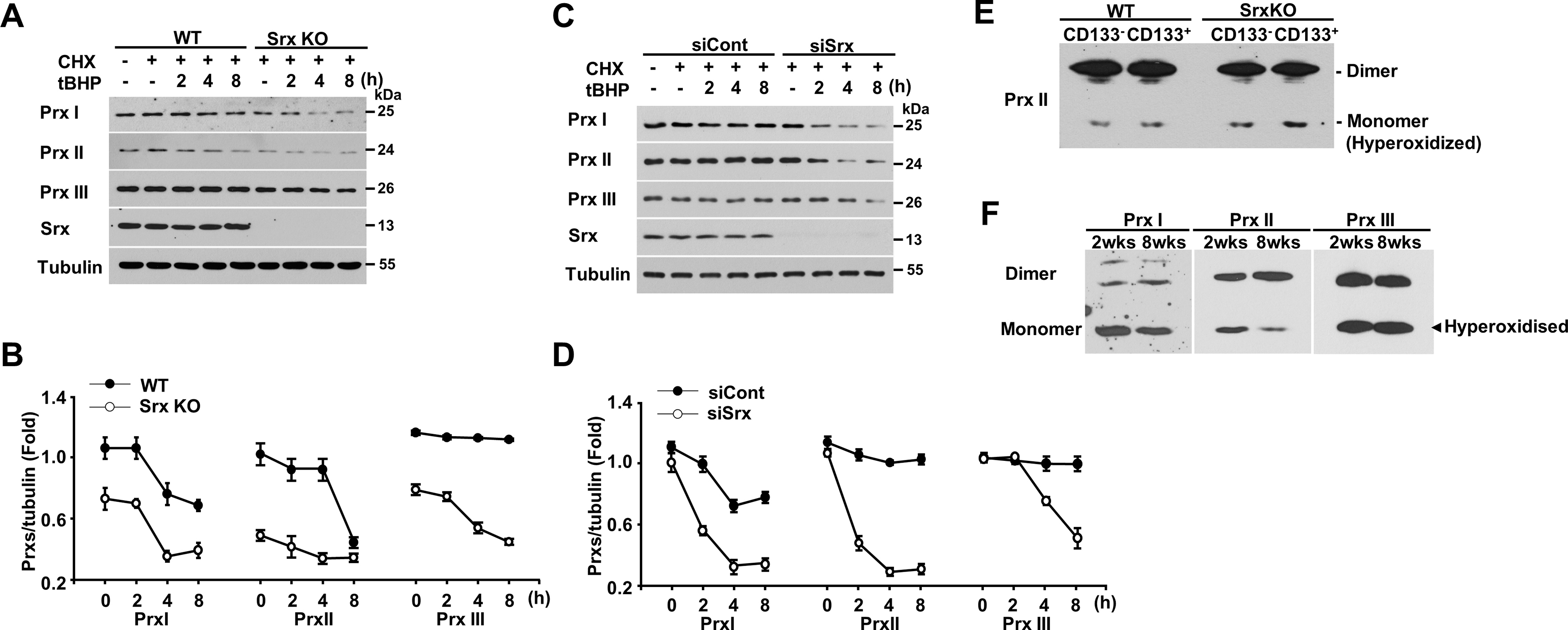

Supplement: figs5 [file mmcfigs5.jpg]

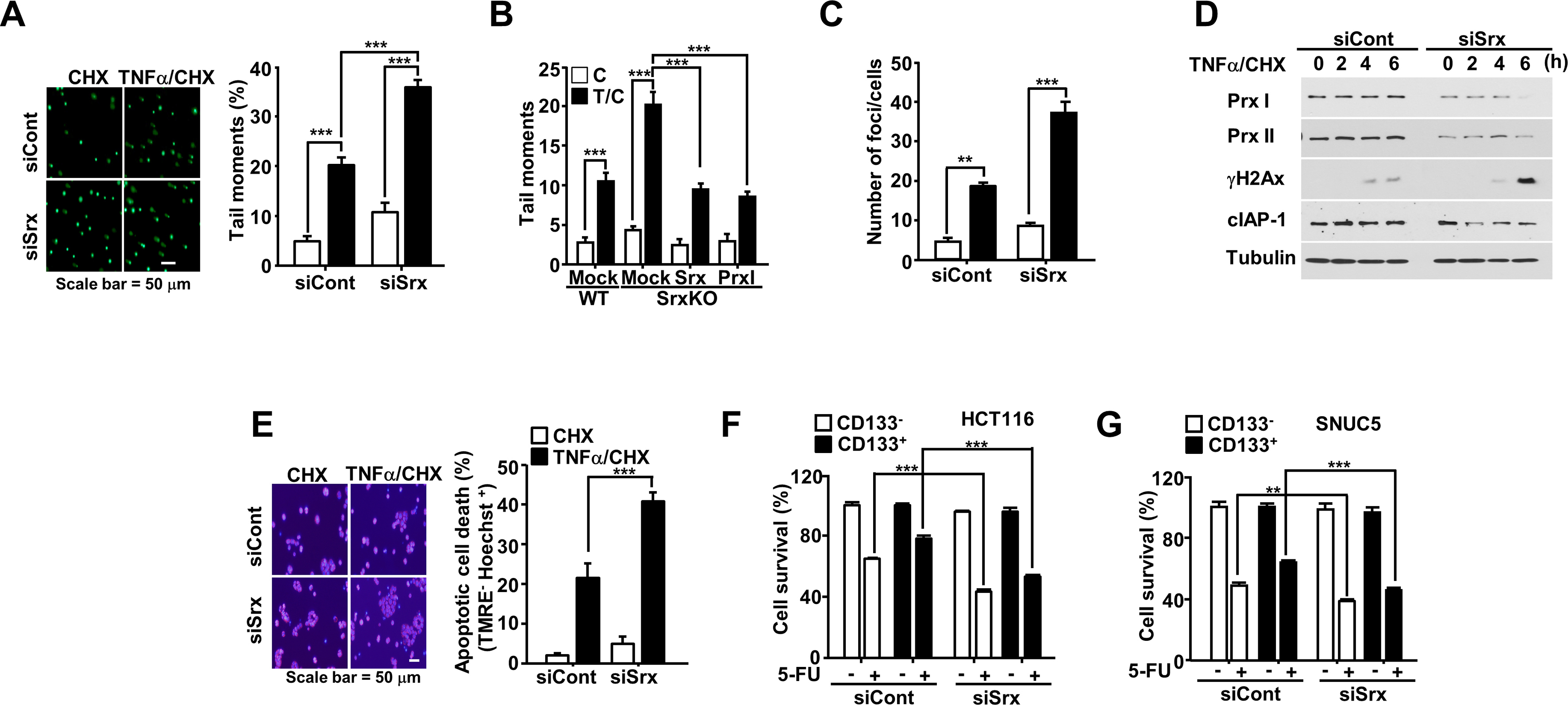

Supplement: figs6 [file mmcfigs6.jpg]
